# A critical review of mathematical models and data used in diabetology

**DOI:** 10.1186/1475-925X-5-43

**Published:** 2006-06-29

**Authors:** A Boutayeb, A Chetouani

**Affiliations:** 1 Department of Mathematics Faculty of Sciences, Oujda, Morocco

## Abstract

The literature dealing with mathematical modelling for diabetes is abundant. During the last decades, a variety of models have been devoted to different aspects of diabetes, including glucose and insulin dynamics, management and complications prevention, cost and cost-effectiveness of strategies and epidemiology of diabetes in general. Several reviews are published regularly on mathematical models used for specific aspects of diabetes. In the present paper we propose a global overview of mathematical models dealing with many aspects of diabetes and using various tools. The review includes, side by side, models which are simple and/or comprehensive; deterministic and/or stochastic; continuous and/or discrete; using ordinary differential equations, partial differential equations, optimal control theory, integral equations, matrix analysis and computer algorithms.

## Introduction

It is now commonly admitted that diabetes is sweeping the globe as a silent epidemic largely contributing to the growing burden of non-communicable diseases and mainly encouraged by decreasing levels of activity and increasing prevalence of obesity [1-5]. Dramatic increase has occurred in both prevalence and incidence of diabetes, especially with the new threshold proposed by the Expert Committee on the diagnosis and classification of diabetes mellitus in 1997 [6] and adopted by the World Health Organization. During the last decades, a huge number of papers were published on different aspects of diabetes and its complications. In particular, an interesting literature has been devoted to studies collecting, analyzing and validating data concerning diabetes populations. A variety of mathematical models, statistical methods and computer algorithms have been proposed in order to understand different aspects of diabetes such as: glucose-insulin dynamics, epidemiology of diabetes and its complications, cost of diabetes and cost-effectiveness of strategies dealing with diabetes. Several reviews have been devoted to mathematical models and diabetes. In their majority, these reviews concentrated on specific aspects of diabetes such as glucose-insulin dynamics [7-13], computer algorithms and devices [14-16], sensors and control [17,18], mathematical and software aspects [19], glycemic index [20], burden and cost of diabetes [21,22]. On the one side, it is difficult to include in one review mathematical models published on different aspects of diabetes. On the other side, it would be very useful for researchers in this field to get a panorama of the models published so-far and their references. The benefit will be greater if such a review is published with free online access, especially for physicians and researchers in developing countries. The challenge is worth trying and the present paper is proposing a non-exhaustive overview including model structure, computer algorithms, data used as input and model validation.

## Glucose-insulin dynamics

### Mathematical models

The majority of mathematical models proposed in the literature were devoted to the dynamics of glucose-insulin, including Intra Venous Glucose Tolerance Test (IVGTT), Oral Glucose Test (OGTT), Frequently Sampled Intravenous Glucose Tolerance Test (FSIGT). In 1939, Himsworth and Ker [23] introduced the first approach to measure the insulin sensitivity *in vivo*. Mathematical models have been used to estimate the glucose disappearance and insulin-glucose dynamics in general. Bolie is among the pioneers in this field. In 1961, using ordinary differential equations, he proposed the following simple model [24]:





where *G *= *G*(*t*) represents the glucose concentration, *I = I*(*t*) represents the insulin and *p*, *a*_1_, *a*_2_, *a*_3_, *a*_4 _are parameters.

Although various models (simple and comprehensive) were proposed by different authors [25-28] (see also the 244 references in the review by Bergman et al. (1985)) [7], especially those dealing with insulin sensitivity, the real start of modeling the glucose-insulin dynamics is thought to begin with the so-called minimal model proposed by the team of Bergman and Cobelli in the early eighties [29,30]. The model was formulated as follows:







where, (*G*(*t*)* - p*_5_)^+ ^= *G*(*t*)* - p*_5 _if *G*(*t*) > *p*_5 _and 0 otherwise.

*X*(*t*) denotes an auxiliary function representing insulin-excitable tissue glucose uptake activity, *G*_*b*_, and *I*_*b *_are the subject's baseline glyceamia and insulinimia. *b*_0 _– *b*_7 _are parameters. It should be stressed that, although equations (3)–(5) were developed for describing FSIGT data, these equations were presented into two parts. Part 1 with equations (3)–(4) and part 2 using equation (5). A large number of papers have been published, using modified versions of the glucose minimal model (3)–(4) for describing OGTT and meal tests, while insulin minimal models derived from (5) are still limited to IVGTT. It should also be stated that the major contribution of the glucose minimal model (3)–(4) has been to provide means of estimating insulin sensitivity *S*_*I *_= *p*3/*p*2, avoiding the glucose clamp.

The same authors have further published papers, completing, testing or validating the results of the minimal model [7,31-33]. An indication of the importance of the minimal model and subsequent research for diabetes understanding is given by the 2006 Banting medal awarded by the American Diabetes Association to Professor Bergman for his achievements.

Variant versions based on the minimal model were considered by different authors. An example of this category was proposed by Derouich and Boutayeb who used a modified version of the minimal model to introduce parameters related to physical exercise [34]:





where *q*_1_, *q*_2_, *q*_3 _are parameters related to physical activity and defined as follows:

*q*_1 _: the effect of physical exercise in accelerating the utilization of glucose by muscles and the liver insulin

*q*_2 _: the effect of physical exercise in increasing the muscular and liver sensibility to the action of insulin.

*q*_3 _: the effect of physical exercise in increasing the utilization of insulin. In other words, *q*_3 _increases insulin effectiveness in enhancing glucose disposal and consequently improving insulin sensitivity to become: *S*_*I *_= (*p*_3 _+ *q*_3_)(1 + *p*_2_)*/P*_2_.

According to a paper published by Bergman in 2002 [35], more than 500 studies related to the minimal model can be found in the literature. More information on this historic model and related models can be found in literature [8,9].

However, some authors [19,36-38] indicated that while the minimal model has minimal number of constants (*p*_0_-*p*_7_), and has been indisputably useful in physiological research, it has the following drawbacks:

1. The model, as originally proposed, is to be regarded as composed of two separate parts. The first part uses equations (3) and (4) and the second part uses equation (5). For the last part, plasma glucose concentration is to be regarded as a known forcing function. In other words, the model parameter fitting has to be conducted in two steps: first, using the recorded insulin concentration as input data in order to derive the parameters in the two first equations, then using the recorded glucose as input data to derive the parameters in the third equation.

2. Some of the mathematical results produced by this model are not realistic(problems of positive equilibrium and solutions not bounded).

3. The artificial non-observable variable *X*(*t*) is introduced to take account of the delay in the action of insulin.

Taking into account these remarks and stressing that the glucose-insulin system is an integrated physiologic dynamical system which should be dealt with as a whole, De Gaetano and Arino [36,37] proposed an aggregated delay differential model called a dynamical model:

After renaming the parameters, the dynamical model takes the form [36]:





with *G*(*t*) = *G*_*b *_for -*b*_5 _≤ *t *< 0

Mukhopadhyay et al. (2004) recalled that this model has been shown to allow simultaneous estimation of both insulin secretion and glucose uptake parameters, to have positive, bounded solutions, and to be globally asymptotically stable around the pre-injection equilibrium blood glucose and insulin concentrations. They proposed an extension by introducing a generic weight function *ω *in the delay integral kernel for the pancreatic response to glucose. The new model obtained is as follows:





with *G*(*t*) = *G*_*b *_for *t *< 0

A more general model was proposed by Li et al. (2001). The authors noted that, while the dynamical model solves the problems of the minimal model, it implicitly or explicitly made a few assumptions that may not be necessary or realistic. Specifically, some of the interaction terms are too special and thus too restrictive. For example, the term *b*_4_*I*(*t*)*G*(*t*) assumes that mass action law applies here while a more popular, general and realistic alternative is to replace this term by *b*_4_*I*(*t*)*G*(*t*)*/*(*αG*(*t*) + 1). The way the delay is introduced is also restrictive. Consequently, the model proposed is the following:





with *G*(*t*) = *G*_*b *_for *-b*_5_*≤ t <*0 and *G*_*t*_(*θ*) = *G*(*t *+ *θ*), *t *> 0, -*b*_5 _≤ *θ *< 0.

Other models of the glucose-insulin dynamics, using optimal control or partial differential equations were proposed by different authors [18,40-44]. Cobelli and Tomaseth [41] discussed the optimal input design in a model of glucose kinetics. They proposed the following model:



*y*(*t*) = *c*(*p*)*x*(*t*)     (15)

*z*(*t*) = *y*(*t*) + *e*(*t*)     (16)

*h *[*x*(*t*), *u*(*t*), *p*] ≥ 0     (17)

Lam et al. [44] used a slightly modified version of the minimal model for the assessment of insulin sensitivity. An interesting survey of mathematical models using control for glucose-insulin and management of diabetes is given by Palerm in his Ph. D thesis, with some 350 references [18]. The author focussed on the Direct Model Reference Adaptive Control (DMRAC) and its reformulation.

The formulation of the general DMRAC algorithm is based on the following system



or more generally,



where *x*(*t*) is the (*nx*1) state vector, *u*(*t*) is the (*mx*1) control vector, *y*(*t*) is the (*qx*1) output vector, *A*, *B*, *C *and *D *are matrices with appropriate dimensions. The objective is to find, without explicit knowledge of *A *and *B*, a control *u*(*t*) such that the output vector *y*(*t*) fellows a reference model.

### Computer algorithms

The regulation of blood glucose concentration is mainly achieved by acting on three control variables: insulin, meals and physical exercise. However, as stressed by Bellazzi et al. [15], the quantitative evaluation of meals and physical effort still represents a major problem in home monitoring. Consequently, the quasi totality of proposed control systems have focused on insulin therapy strategies. A number of devices-microsystems and computer approaches- have been reported in the literature with open, closed and partially closed algorithms, Selam and Charles [45], Lehman and Deutsch [46,47]. Two reviews on the intravenous route to blood glucose control and subcutaneous route to insulin dependent diabetes therapy were recently published by Bellazzi et al. [15] and Parker et al. [16]. A partial list of software packages, commercially and freely available is given in a recent review by Makroglou et al. [19].

### The closed-loop strategy

An example of models with closed-loop strategy is a wearable artificial pancreas as proposed by Shimoda et al. [18,48], based on the assumption that the relationship between plasma insulin and blood glucose concentration in a normal subject during an oral glucose bolus is as follows:



where *I *denotes the plasma insulin concentration; *G *is the blood glucose concentration; and *a*, *b *and *c *are parameters that can be estimated by nonlinear least squares method [49].

The insulin dynamics is described by the following ordinary differential system:









where *V *is the plasma volume, *IIR *represents the Insulin Infusion Rate; *X*, *Y *and *Z *are the insulin masses in the two subcutaneous compartments and in plasma respectively.

### The open-loop approach

According to the review by Bergman et al. [7], two distinct classes of methodologies were applied to open the glucose/insulin feedback relationship *in vivo*. The first method pioneered by Reaven and his colleagues [50], and labelled as the pancreatic or insulin suppression test, utilized pharmacologic means to render the pancreas blind to plasma glucose concentration. The second approach, labelled as the glucose clamp, was proposed by Andres [51]. The method uses a variable glucose infusion to establish a relatively constant plasma glucose concentration with or without exogenous insulin. Mathematical models are also proposed to deal with the deterioration of beta-cell [52-54].

### Epidemiological models applied to diabetes

Historically, since the first model of smallpox formulated by Bernoulli in 1760, an abundant literature was devoted to mathematical models dealing with communicable diseases such as measles, rubella, malaria, influenza, AIDS, dengue and others [56]. As indicated by a review published by Hethcote in 2000 [57], a tremendous variety of models have been formulated, mathematically analyzed, and applied to infectious diseases. Modelling has thus become an interesting tool providing conceptual results such as thresholds, basic reproduction numbers, contact numbers, and replacement numbers. Application of similar models for non communicable diseases is rather unusual. In this way, few authors have proposed epidemiological models for diabetes and obesity [58-66]. In [59], Boutayeb and Derouich considered two discrete models for the evolution from diabetes without complications to the stage of diabetes with complications.

In [60], using partial differential equations, the authors proposed an age structured continuous model for complications of diabetes. Supposing that *C *= *C*(*a*, *t*) and *D *= *D*(*a*, *t*) represent the numbers of diabetics with and without complications aged *a *at time *t*, respectively, and *n*(*a*, *t*) = *C*(*a*, *t*) + *D*(*a*, *t*) the size of the population of diabetics aged *a *at time *t*, different scenarios with different values were used for the following parameters: natural death rate (*d*(*a*, *t*)), death rate due to complications (*δ*(*a*, *t*)), incidence of diabetes with and without complications (*I*_1_(*a*, *t*)), (*I*_2_(*a*, *t*)) rate at which complications are developed (*p*(*a*, *t*)) and rate at which complications could eventually be cured (*q*(*a*, *t*)). The main objective of the authors was to show that, although diabetes is not curable at the moment, prevention of its complications (which is possible) would improve peoples quality of life and reduce costs of the national health and social services. Assuming that the number of males is equal to the number of females and that diabetes affects the people of the two sexes equally, the continuous age structured model is formalized by the following partial differential equations:





adding equations (25) and (26) and writing :

*C*(*a*, *t*) = *r*(*a*, *t*)*n*(*a*, *t*)     (27)

leads to



In the same spirit, Boutayeb and colleagues [62,63] proposed linear and non-linear population models of diabetes mellitus, using ordinary differential equations and numerical implementation.

## Data analysis, parameters estimation and validation

### Studies and trials

Worldwide, different studies were devoted to diabetes and its complications. These studies have been used directly or indirectly for data analysis, mathematical modeling and parameters validation. Among the most cited studies, Diabetes Control Complications Trial (DCCT) [67] and UK Diabetes Prevention Study (UKPDS) [68]. The first trial involved 1,441 volunteers with type 1 diabetes and 29 medical centers in the United States and Canada, and have shown that, diabetes complications can be reduced or at least delayed by a good regular glycemic control through intensive insulin therapy consisting of three or more insulin injections per day or in the use of insulin pumps. The main DCCT Study Findings were the following: Lowering blood glucose reduces the risks of eye disease, kidney failure and nerve disease by 76%, 50% and 60% respectively [69-72]. The second trial concerned over 5000 non insulin-dependent patients from 23 centres from all parts of England, Scotland and Northern Irland, showing that complications of diabetes can be prevented by a better control of blood glucose and blood pressure. Among the 70 papers published by the UKPDS group, we cite here some of those using mathematical models. The UKPDS risk engine was used as a model for the risk of coronary heart disease, myocardial infarctus and stroke in Type II diabetes [73-75]. Modeling glucose exposure as a risk factor for photocoagulation in Type II diabetes was considered in [76], whereas the UKPDS Outcomes Model was proposed to estimate the lifetime health outcomes of patients with Type 2 [77]. Data collection, parameters estimation and validation concerned a multitude of other studies such as: the Wisconsin epidemiologic study of diabetic retinopathy (WESDR) [78], Framingham Heart Study (FHS) [79], Diabetes Prevention Program (DPP) [80], Health Outcomes Prevention Evaluation (HOPE) [81,82], The Health Plan Employer Data and Information Set (HEDIS) [83], Echantillon national temoin représentant des personnes diabétiques (ENTERED) [84], Multiple Risk Factor Intervention Trial (MRFIT) [85], Heart Protection Study (HPS) [86], Cholesterol and Recurrent Events (CARE) [87], ACE Inhibitors and Diabetic Nephropathy Trial (Lewis) [88], the IRMA-2 trial [89], Irbesartan Diabetic Nephropathy Trial (IDNT) [90], the Collaborative AtoRvastatin Diabetes Study (CARDS) [91].

### The archimedes model

According to [92], until recently, there have been four main kinds of mathematical models in health care:

1. Biological modeling,

2. Clinical Medicine,

3. Operations research,

4. Economic/system resources.

Thus, the authors present Archimedes as a new type of mathematical model which includes all four components. Archimedes is a very detailed, comprehensive, continuous simulation model. A person-by-person, object-by-object simulation, spanning from biological details to the care processes, logistics, resources, and costs of health care systems [92-94]. The model is written in differential equations for which different levels of detail may be considered [95]. The equations, assumptions, and sources are summarised in an online appendix (available at ). A validation of the Archimedes model of diabetes and its complications or a variety of populations, organ systems, treatments, and outcomes is given by Eddy & Schlessinger [96,97]. The model was validated against 18 trials of which ten trials explicitly dealing with diabetes. Namely: the Diabetes Control and Complications Trial(DCCT) [67], the U.K. Prospective Diabetes Study (UKPDS) [68], the Diabetes Prevention Program (DPP) [80], the Health Outcomes Prevention Evaluation (HOPE) [81], the diabetes sub-population of the HOPE Trial (Micro-HOPE) [82], the Heart Protection Study (HPS) [86], Cholesterol and Recurrent Events (CARE) [87], the ACE Inhibitors and Diabetic Nephropathy Trial (Lewis) [88], the IRMA-2 trial [89], and the Irbesartan Diabetic Nephropathy Trial (IDNT) [90]. In general, between 10 and 30 equations are needed to represent the pathophysiology of the disease and calculate the effect of a specific treatment on a specific outcome in a specific population (not included equations for behaviors, care processes, logistics, and other nonbiological aspects of the model. As stressed by the Editorial of Diabetes Care [98], functional forms of the equation are given but values of the variables and parts of the model that describe micro- and macro-vascular complications are not provided. However, beyond these limitations, the model was used to predict 74 major outcomes, giving astounding results: In 71 out of the 74 clinical outcomes, the differences between the results calculated by the model and the observed ones were statistically not significant. More information on Archimedes model can be found in [92].

### Other models

Other models and computer algorithms were devoted to the burden, cost and cost-effectiveness of diabetes [99-101], telemedicine and home management of diabetes [102-104]. In a case study paper [104], Wu proposed the following model for self-management of Type 2 diabetes:



where *x *represents blood glucose level over the baseline at time *t *and *ω*_0 _is the system natural frequency. Finally, in some papers and letters, mathematical models and guidelines for computer modeling of diabetes were subject to debate and criticism [105-107].

## Discussion

Mathematical models constitute interesting tools for the understanding of diseases. They provide insights, improve intuitions, clarify assumptions for formal theory, allow for planning studies, estimating parameters, determining sensitivities, assessing conjectures, simulating simple and complex phenomena and providing future predictions. In the case of diabetes, simple and comprehensive models dealing with different aspects of the disease, have been used during the last three decades. In general, simple models are so simple as to be inadequate but they have the advantage of using a small number of identifiable parameters. Comprehensive models on the other hand are models which try to represent the system (biological, clinical, economic, etc...) by taking into account all interactions. This makes them very complex and generally not identifiable. In the present paper, our main objective was an overview of models and studies dealing with different aspects of diagnosis, care and management of diabetes and its complications. We presented a non exhaustive list of published models with their theoretical and applied aspects, indicating what were the hypotheses that lead authors to propose new, modified, generalized or alternative models. But we must say that we did not intend to compare all models or classify them according to whatever criterion of performance. Even when available models are simple, they are not necessary comparable. For instance, Bolie's model [24] is one of the simplest models proposed to estimate the linear glucose disappearance and insulin-glucose dynamics. However, since the author is one of the pioneers in this field, his work remains an unavoidable reference. Another very simple model was recently proposed in a case study by Wu [104] but the purpose was a self-management of type 2 diabetes. The model being a case study based on a single type 2 diabetes person, the results yielded need to be considered with caution. The discrete matrix model considered by Boutayeb and Derouich [59] is also simple but the approach is completely different since it deals with the control of complications Bergman et al. [7] discussed seven models before selecting the " best one" which became from then the well known minimal model (between simple inadequate and comprehensive not identifiable models), they based their selection on the following criteria:

1. to be physiologically based,

2. having parameters that can be estimated with a reasonable precision,

3. parameters with values that are reasonable and have physiological interpretation,

4. best able to simulate the dynamics of the system with smallest number of identifiable parameters.

In section 2, variant versions of the minimal model were considered by different authors. For instance, in Derouich and Boutayeb [34], physical exercise was seen to be an interesting tool that improves insulin sensitivity (*S*_*I *_= (*p*_3 _+ *q*_3_)(l + *p*_2_)/*p*_2_)). The authors stressed that new control strategies take a long time before they become affordable on a large scale, especially in developing countries where the majority of diabetics are struggling just to get insulin doses and where the price of a blood strip exceeds the individual income.

As indicated earlier, the minimal model has been indisputably useful in physiological research and served as starting point for many other models. The drawbacks raised by De Gaetano and Arino [36] were mainly based on the mathematical formulation (specifically problems of positive equilibrium and solutions not bounded). But they stressed that no criticism is implied regarding the practical usefulness of the minimal model. By the way, they acknowledge that their group uses the minimal model in the routine evaluation of insulin sensitivity in clinical patients. The so-called "dynamic model" proposed by the previous authors has not escaped from criticism since the assumptions made were judged to be not necessary or realistic. This judgement was made by Li et al. [38] who also criticised the restrictive way of using the delay and proposed a more general model. According to these authors, their general model was constructed for the study of IVGTT which focuses on the metabolism of glucose. However, except simulation and mathematical aspects (steady state, oscillatory glucose and insulin levels), no evidence is given on the real performance. For computer algorithms, as stressed earlier, the quasi totality of proposed control systems have focused on insulin therapy strategies. In many cases, the proposition of models are dictated by commercial purposes and the accessibility to their pragmatic use remains restrictive. Finally, among complex comprehensive models, Archimedes model can be seen as the most illustrative. The huge arsenal of computer and mathematical tools used by this model seems to be justified by the first validated results. However, the model has been published recently and more time is needed to get sufficient information for a critical discussion.

## Conclusion

During the last decades, an interesting number of papers have been published on mathematical models and computer algorithms. In the present review, the authors have tried to give a non-exhaustive panorama of the papers which have used mathematical modeling for different aspects of diabetes, including glucose-insulin dynamics, beta-cell function, epidemiology of diabetes, management and the burden of diabetes and its complications.

As indicated in section two, the award of 2006 banting medal by the American Diabetes Association to Professor Bergman for his achievements in diabetes research among which, the famous minimal model, is an indication of the importance of mathematical models for the understanding of diabetes and its management.
